# Advantages of Slow Sensing for Ambient Monitoring: A Practical Perspective

**DOI:** 10.3390/s23218784

**Published:** 2023-10-28

**Authors:** Juan Casanova-Chafer

**Affiliations:** Chimie des Interactions Plasma Surface, Institute for Materials Science and Engineering, Université de Mons, Place du Parc 23, 7000 Mons, Belgium; juan.casanovachafer@umons.ac.be

**Keywords:** slow sensing, ultrafast sensors, resistive sensors, ambient monitoring, limit of detection

## Abstract

Air pollution is a ubiquitous threat, affecting 99% of the global populace and causing millions of premature deaths annually. Monitoring ambient air quality is essential, aiding policymakers and environmental agencies in timely interventions. This study delves into the advantages of slower gas sensors over their ultrafast counterparts, with a keen focus on their practicality in real-world scenarios. Slow sensors offer accurate time-averaged exposure assessments, harmonizing with established regulatory benchmarks. Their heightened precision and reliability, complemented by their cost-effectiveness, render them eminently suitable for large-scale deployment. The slow sensing ensures compatibility with regulations, fostering robust risk management practices. In contrast, ultrafast sensors, while claiming rapid detection, despite touting swift detection capabilities, grapple with formidable challenges. The sensitivity of ultrafast sensors to uncontrolled atmospheric effects, fluctuations in pressure, rapid response times, and uniform gas dispersion poses significant hurdles to their reliability. Addressing these issues assumes paramount significance in upholding the integrity of air quality assessments.

## 1. Introduction

According to the World Health Organization (WHO), 99% of the world’s population is subjected to air pollution levels that exceed the WHO’s recommended thresholds. This prolonged exposure, typically imperceptible and often devoid of odor, gives rise to grave health concerns, including acute and chronic respiratory ailments, strokes, cardiovascular diseases, and even lung cancer [1]. Consequently, the WHO accounts for an alarming 6.7 million premature deaths each year [2].

Continuous monitoring of ambient air quality is essential to preventing exposure to harmful concentrations of airborne pollutants. These pollutants, ranging from particulate matter to volatile organic compounds, have been unequivocally linked to a variety of health issues [3] and adverse environmental consequences [4], including the emission of greenhouse gases that drive climate change. The sources of air pollutants vary widely, encompassing large-scale industrial activities leading to outdoor pollution, such as the combustion of fossil fuels, and commonplace household tasks contributing to indoor contamination, like cooking, which releases pollutants during kitchen combustion processes [5]. In homes lacking adequate ventilation, this can pose a significant issue.

By constantly tracking pollutant levels, we gain real-time insight into potential health hazards, allowing policymakers, governmental institutions, and environmental agencies to implement timely interventions and protective measures [6]. In this context, the significance of deploying an extensive sensing network with a high density of measuring nodes to monitor ambient pollution is crucial [7,8]. Due to the minute scale of pollutant variations, a dense network can capture localized sources and fluctuations, enabling precise identification of pollution hotspots [9]. However, achieving such high node density necessitates affordable sensors capable of real-time monitoring. 

Among the array of available technologies, chemical resistive (chemiresistive) sensors have garnered attention as a promising choice for ambient monitoring [10]. Their appeal lies in their cost-effectiveness, straightforward readouts, durability, scalability for large-scale production, potential miniaturization, and ease of integration into monitoring systems [11,12]. Consequently, this perspective primarily focuses on chemiresistors, which align with the prerequisites for effective ambient monitoring. However, it should be noted that a notable limitation of chemiresistors is the operating temperature, especially concerning metal oxide-based sensors. These devices often require high temperatures, leading to increased costs due to the introduction of heating elements and higher power consumption [13]. Additionally, continuous operation at elevated temperatures can potentially compromise the properties of the sensitive layer [14]. To mitigate this issue, there is ongoing research to develop nanomaterials capable of functioning at room temperature. However, such materials often exhibit reduced sensitivity [15]. 

Another challenge with chemiresistors is their limited selectivity. The nanomaterials often lack specificity for compounds, resulting in cross-sensitivity with other substances in the atmosphere [16]. One approach to addressing this limitation involves the development of sensor arrays, combining different nanomaterials with varying sensitivities to target gases [17]. While this solution offers improved selectivity, it also introduces complexity and increases device costs.

There is a growing trend in the field of gas sensors, with a focus on developing “ultrafast sensors” that detect pollutants within mere seconds or milliseconds [18,19,20,21]. These ultrafast sensors, characterized by their rapid response times, have become a focal point in research, seemingly driven by a competition to achieve the fastest detection speeds. However, it is essential to consider that established exposure thresholds for pollutants often span from hourly to daily and even yearly averages. In this context, we can define “slow sensors” as devices capable of providing reliable measurements within the range of minutes, aligning well with governmental and market requirements. Amidst the rush for ultrafast sensors, these slower but accurate sensors are sometimes overlooked, despite their ability to meet essential monitoring standards. For instance, European air quality standards specify 1 hour as the shortest averaging period for NO_2_. Consequently, if our slower sensor can measure pollutant concentrations every 5 min, it will yield up to 12 measurements within this averaging period, likely sufficient for accurate averaging.

It can be worth considering an intermediate category; sensors falling within this range can detect pollutants in a matter of tens of seconds. At this juncture, it is imperative to underscore the diverse needs of gas sensors contingent upon their applications. The rapid detection of sudden spikes of potentially life-threatening gases (e.g., chemical warfare agents or radioactive gases) is critical [22]. However, in most scenarios, the nature and concentration of pollutants released into the atmosphere do not pose an immediate threat to life. Therefore, for ambient monitoring of gases like SO_2_ and NO_X_, ultrafast sensors are not a requisite [23].

The main objective of this manuscript is to delve into the rationale behind the superiority of slower gas sensors for ambient monitoring. By focusing on slow sensing, this study endeavors to elucidate their advantages over ultrafast sensors and underscore their potential for furnishing more precise and reliable measurements in ambient monitoring settings. Through an exploration of the benefits associated with slower response times, this research aims to contribute to the ongoing discourse surrounding the selection of gas sensor technology for effective ambient monitoring practices.

## 2. Advantages of Slow Sensing

For better comprehension, the benefits of employing slow sensors in front of ultrafast sensors are divided into subsequent sections. Various factors are explored to demonstrate that slower gas sensors are better suited to meet societal and market demands for ambient monitoring as opposed to their ultrafast counterparts.

### 2.1. Time-Averaged Exposure Assessments

Threshold-limit exposures are critical in determining the safe levels of gas concentrations for human exposure over specified time periods. These limits, established by regulatory bodies and scientific organizations, account for factors such as toxicity, cumulative effects, and exposure duration. These are typically defined for periods of 8 h (working day), 24 h (daily exposure), or even annually [24,25]. These threshold limits are usually specified as recommended and permissible exposure limits (REL and PEL, respectively). Table 1 summarizes prominent regulatory agencies and organizations responsible for setting gas exposure limits in various countries in 2023.

Slow gas sensors, meaning measurements over a few minutes, are instrumental in delivering more representative data for long-term exposure assessments. By capturing over a few minutes, these sensors facilitate the smoothing out of short-term fluctuations in gas concentrations. Transient variations in ambient gas levels, stemming from factors such as wind patterns, weather conditions, human activities, or industrial processes, can lead to temporary spikes or drops in gas concentrations. However, these short-term fluctuations may not necessarily reflect the overall exposure levels that are relevant to the established threshold limits. In contrast, ultrafast sensors are highly susceptible to uncontrollable fluctuations, potentially resulting in false–positive readings. 

The inherent averaging effect of slower sensors ensures that the measurements offer a more accurate representation of average gas concentrations over a specified period. This methodology aligns with the principle of time-averaged exposure assessments, providing a more precise portrayal of potential risks associated with gas pollutants in the ambient environment. By minimizing the impact of momentary spikes or dips, slow sensors contribute to a clearer understanding of long-term exposure levels. Consequently, slow sensing enables more dependable assessments of the health and environmental implications linked to specific air pollutants. 

Furthermore, by prioritizing time-averaged measurements, slow sensors facilitate enhanced risk management strategies. Decision-makers can focus interventions or mitigation efforts based on comprehensive data that accounts for average exposure levels rather than reacting excessively to short-term fluctuations. This approach ensures that resources and efforts are allocated in a targeted manner to address the real risks associated with long-term exposure, rather than temporary variations in gas concentrations.

### 2.2. Enhanced Accuracy and Reliability

When considering the accuracy and reliability of gas sensors for ambient monitoring, a comparison between slow and ultrafast sensors reveals significant disparities in their susceptibility to false positives, interferences, signal-to-noise ratios, and sensitivity to environmental variables. F. Chraim et al. demonstrated the efficacy of increasing the window size (cumulative time for gathering counts of detection) approximately sixfold in a monitoring network with 20 wireless devices designed to detect propane leaks in industrial environments [26]. By employing this approach, the authors observed a fourfold reduction in false positives. This enhancement in sensing performance inherently improves various parameters, including sensor accuracy, enabling the reliable detection of pollutant concentrations.

Slow gas sensors tend to exhibit lower susceptibility to false positives and interferences compared to their ultrafast counterparts. Ultrafast sensors, due to their rapid response times, may be more susceptible to variations in temperature, humidity, pressure, and other environmental parameters. These variations can distort sensor responses, leading to inaccuracies in measurements. Consequently, ultrafast sensors may yield false positive readings, misinterpret data, and trigger unnecessary alarms. In contrast, slow sensors, with their extended response times, offer stability by averaging out these transient fluctuations, thereby reducing the likelihood of false positives and minimizing sensitivity to environmental factors. The enhanced accuracy and reliability of slower gas sensors play a vital role in ambient monitoring, offering measurements less prone to inaccuracies and interferences, thus enhancing data interpretation and decision-making. 

**Figure 1:**
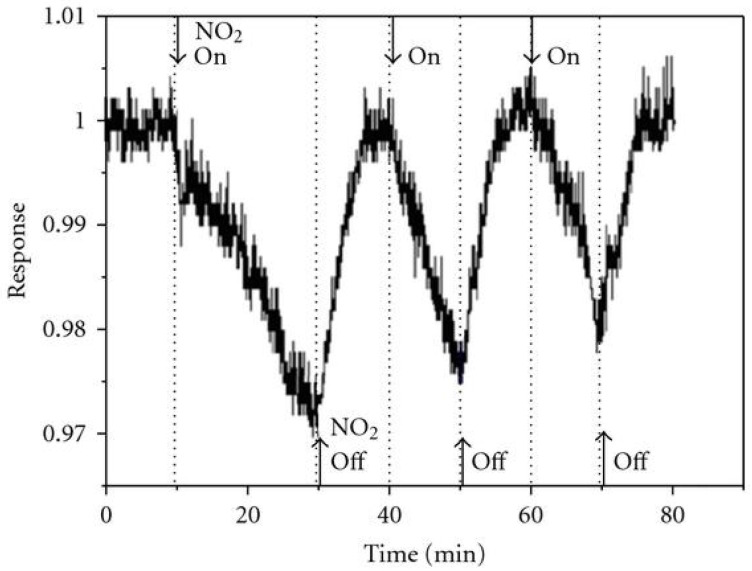
Sensing response of carbon nanotubes (CNT) to three consecutive pulses of 50 ppb of NO_2_. Reproduced from [27], with permission from Elsevier.

Additionally, slow sensing generally exhibits improved signal-to-noise ratios. The extended measurement duration enables comprehensive signal sampling and averaging, resulting in a stronger signal relative to background noise. This improved signal-to-noise ratio enhances measurement accuracy and reliability, reducing uncertainties and mitigating the impact of random environmental fluctuations. For example, Figure 1 illustrates a noisy measurement, even within a minute range, when detecting three times 50 ppb of NO_2_ [27]. Consequently, the accuracy and repeatability of these measurements become challenging. The signal-to-noise ratio is generally associated with inherent device properties and the system setup. Thereby, given that a higher exposure time typically results in an increased response for a given signal-to-noise ratio, this heightened signal change, amidst stable noise levels, inevitably improves the overall signal-to-noise ratio [28,29]. In the case of ultrafast sensors, this issue can be exacerbated; a reduction in exposure time leads to smaller responses, complicating the correct accuracy and reliability of the sensing results.

### 2.3. Cost-Effectiveness and Implementation

When considering the implementation of gas sensors for ambient monitoring, an analysis of resource allocation and cost implications is essential. Comparing the cost-effectiveness and ease of implementation between slow and ultrafast gas sensors provides valuable insights into their suitability for large-scale monitoring networks.

Compared to ultrafast sensors, slower sensors often feature simpler designs and require less sophisticated technology. This simplicity translates into lower manufacturing costs, making slower sensors more affordable and accessible for widespread deployment. The cost-effectiveness of slower sensors enables the establishment of extensive monitoring networks, covering a broader range of monitoring sites and providing a more comprehensive understanding of ambient gas concentrations [30].

Furthermore, the implementation and maintenance of slower sensors are typically more straightforward compared to ultrafast sensors. The slow sensors can be easily integrated into existing monitoring infrastructure, leveraging established monitoring protocols and frameworks. Additionally, their longer response times allow for less stringent real-time data processing requirements, reducing the complexity and cost of the monitoring system [31]. Carbon-based nanomaterials, such as graphene and carbon nanotubes (CNTs), or the utilization of molecular compounds like phthalocyanines and thiols, are typically operated at room temperature due to their inherent properties [32,33]. It is noteworthy that room-temperature operation generally results in relatively longer response times, which can limit their application in ultrafast gas sensors. Nevertheless, when these nanomaterials are operated at room temperature, they find utility in slower sensors, where longer exposure times are acceptable. 

Operating at room temperature holds significant importance, offering a pathway to enhanced cost-effectiveness. For instance, a slow sensor functioning under these conditions can eliminate the need for a heating element, reducing both the economic cost associated with this component and the overall device complexity. Lower operating temperatures also translate to reduced power consumption, a critical parameter in contemporary sensor technology. Furthermore, slow sensors offer extended device lifetimes in comparison to fast-sensing devices operated at higher temperatures, adding to their economic appeal.

Moreover, the reduced complexity and lower cost of maintenance for slower sensors contribute to their long-term viability. Maintenance requirements, such as calibration, sensor replacements, and data management, are generally more manageable and cost-effective for slower sensors [34]. This ensures sustainable and ongoing monitoring efforts, guaranteeing the continuity and reliability of data collection over extended periods.

### 2.4. Compatibility with Regulations and Guidelines

Air quality and workplace safety are governed by a variety of regulations and guidelines established by regulatory bodies. These regulations define PELs and RELs for various air pollutants to ensure the protection of public health and occupational safety [35]. The compatibility of gas sensors, particularly slow sensors, with these regulations and guidelines is a crucial aspect to consider in ambient monitoring.

Regulations related to ambient air quality typically specify time frames for assessing gas concentrations. For instance, the main regulatory institutions worldwide define the 1 h exposure as the shorter time frame, along with the typical working day (8 h), daily (24 h), and annual periods. The slow sensing aligns well with these time frames. The longer response times enable measurement collection over several minutes, rendering them suitable for evaluating gas concentrations within the specified time periods. By providing averaged measurements, slower sensors adhere to the principles of time-averaged exposure assessment, the foundation for establishing threshold limits in many regulations. 

The compatibility of slow sensing with regulations and guidelines ensures that the monitoring data obtained directly corresponds to the prescribed limits. This facilitates compliance monitoring and enables accurate assessments of potential risks and exposures. Decision-makers, environmental agencies, and employers can confidently rely on slow sensors to provide measurements that align with the specified time frames and averaging principles, ensuring that monitoring efforts are consistent with regulatory requirements. Moreover, the compatibility of slower sensors with regulations allows for easier integration of monitoring data into risk management strategies and mitigation efforts. The collected data can be directly compared to the established limits, facilitating the identification of areas where action is required to ensure compliance and protect public health or occupational safety.

It is worth considering a practical scenario. If we require a gas sensor to monitor the shortest time frame (1 h exposure), two options are available. The first is an ultrafast sensor claiming detection capability within 5 s. This implies that within each 1 h averaging period, the ultrafast sensor provides 720 measurements with relatively low accuracy. Conversely, the second option is a slow sensor, measuring every 5 min, requiring 60 times the duration of the previous sensor to obtain a single measurement. However, this slower approach yields 12 measurements within 1 h, offering greater reliability and accuracy. Despite the abundance of research advocating for ultrafast sensors, particularly in laboratory conditions, the second option proves more realistic and practical for real-world applications. 

### 2.5. Limitations of Slow Sensing

It is also important to acknowledge some limitations of slow sensors, particularly when compared with faster alternatives. The main limitation is probably based on the relatively lower sensitivity to sudden spikes in gas concentrations. Longer response times in slow sensors can result in reduced sensitivity to abrupt increases in gas levels. This characteristic might be a drawback in situations requiring immediate detection of acute exposure threats, such as chemical warfare agents or radioactive gases, where rapid response is paramount. Consequently, in emergency scenarios demanding swift detection due to the potentially hazardous effects of the gas pollutant within seconds, slow sensing may not be the most suitable choice. This balanced perspective highlights the nuanced considerations between slow and ultrafast sensors, emphasizing the contextual nature of their effectiveness in different applications.

## 3. Experimental Issues and Challenges of Ultrafast Sensors

When examining the precision and trustworthiness of gas sensors for ambient monitoring, a comparison between slow and ultrafast sensors exposes significant challenges and misinterpretations that are frequently overlooked. Two key issues are highlighted to emphasize the concerns associated with ultrafast sensing.

### 3.1. Misleading Detection and Quantification

A fundamental problem often seen in gas sensor research is the confusion between detection and quantification [36]. Detection, assessed by the limit of detection (LOD), signifies the ability to identify the presence of a specific gas pollutant without determining its concentration. Quantification, on the other hand, involves precisely measuring the gas concentration, usually indicated by the limit of quantification (LOQ).

Researchers tend to focus on detection while neglecting the complexities of quantification. Ultrafast sensors face challenges related to the signal-to-noise ratio due to their rapid response times. Accurate quantification demands a signal significantly higher than the noise level, which becomes difficult for ultrafast sensors with minimal exposure times. Rapid responses require a delicate balance between capturing an adequate signal and avoiding noise interference [37].

Neglecting LOQ considerations can lead to erroneous conclusions. While a sensor might successfully detect a specific gas, the absence of quantification complicates assessing the actual risk or comparing it against regulatory standards. This oversight can result in inaccurate risk assessments, potentially impacting public health policies and environmental regulations.

To rectify this issue, researchers must not only focus on a sensor’s ability to detect specific gases but also emphasize quantification. Incorporating LOQ calculations into methodologies is crucial. Additionally, it is essential to clearly distinguish between detection and quantification limits in research papers. Acknowledging these limitations and discussing the associated challenges would lead to more nuanced and accurate interpretations of sensor data. This approach ensures that research findings are not only scientifically rigorous but also practically applicable, aiding policymakers and stakeholders in making well-informed decisions regarding air quality and pollution control measures.

**Figure 2:**
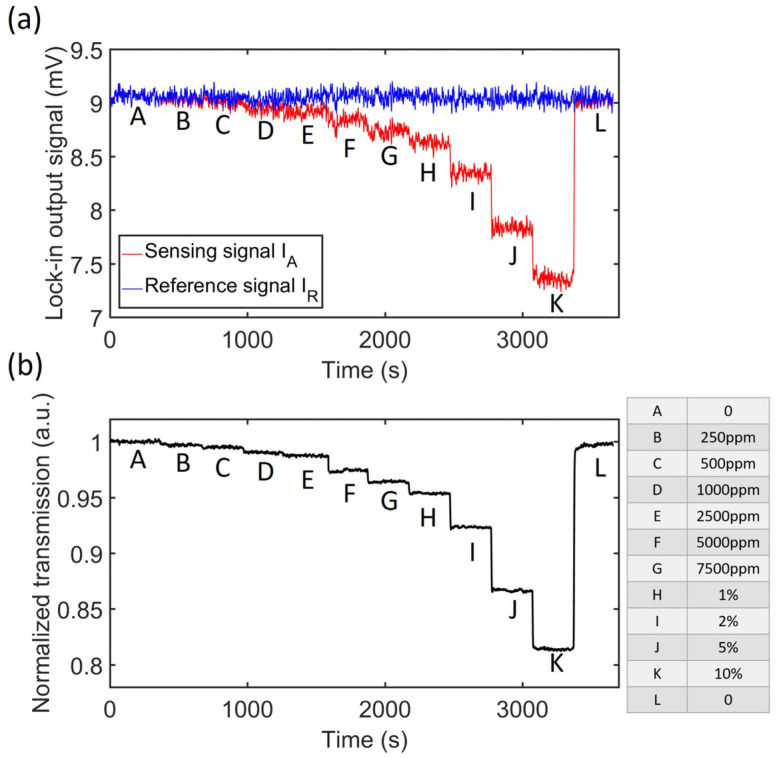
(**a**) Sensing and reference signals for different CO_2_ concentrations. (**b**) Normalized response. The table summarizes the CO_2_ concentrations applied for every step. Reproduced from [38], with permission from MDPI.

Figure 2 depicts a scenario where researchers claim their sensors have a limit of detection of 100 ppm with a response time of 2.8 s [38]. However, the graphs show noisy measurements and reduced responses, especially for the lowest concentrations tested. Considering the subtle change in stage B (corresponding to 250 ppm of CO_2_), it is unclear if this sensor can accurately measure significantly lower concentrations, such as 100 ppm.

**Figure 3:**
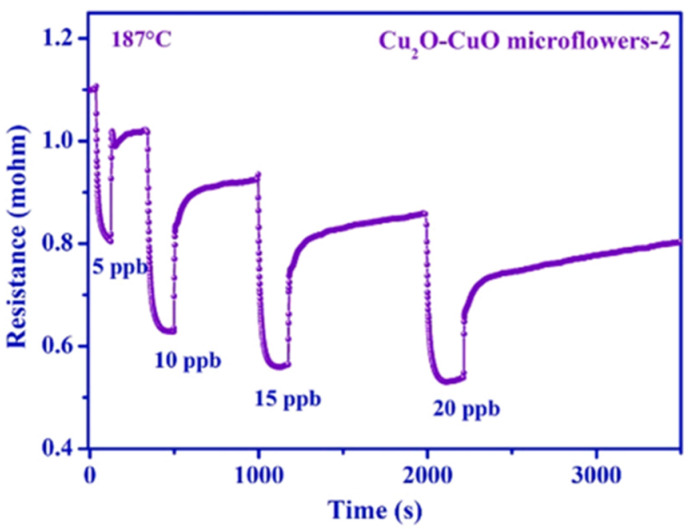
Sensing response employing Cu_2_O–CuO microflowers for detecting several concentrations of NO_2_ at 187 °C. Adapted from [39], with permission from Elsevier.

Additionally, it is worth mentioning that while LOD and LOQ are vital parameters to evaluate gas sensor performance, there are common mistakes researchers make:

(a) **Underestimating the LOD**: Many studies state their LOD corresponds to the lowest concentration they experimentally tested. However, if a specific gas concentration is detected with a substantial response and low noise, the LOD value should be lower than this lowest concentration. Researchers often underestimate their LOD values, as in the case presented in Figure 3. In this work, the researchers claim that their LOD is 5 ppb [39]. However, upon analyzing the graph, it becomes evident that this concentration represents the lowest value they practically monitored. Regrettably, due to the noteworthy signal-to-noise ratio and the regression analysis involving various concentrations, these authors are undervaluing the LOD of their nanomaterial.
(b) **Importance of LOQ**: While determining LOD is crucial to ascertaining a sensor’s ability to detect a specific gas pollutant, from a practical standpoint, LOQ holds greater significance. LOQ reveals the lowest concentration a sensor can reliably detect. Unfortunately, few works consider this essential sensing parameter [40,41] vital for commercial applications.
(c) **Calculation of LOD and LOQ values**: Traditionally, determining the LOD and LOQ involves applying signal-to-noise (S/N) ratios of 3 and 10, respectively. While this method is widely adopted, it often leads to overestimated values. The issue stems from the fact that only an isolated measurement of a specific concentration is considered, neglecting the complexities of real-world scenarios. A more accurate method for their determination involves considering multiple concentrations of the target pollutant and different measurement cycles [42] of each concentration (repeatability), which is often overlooked. This method, utilizing Equations (1) and (2), accounts for calibration curves and standard deviations between measurements, providing more reliable results.

(1) $$\mathrm{LOD}=\frac{3S_{a}}{b}$$

(2) $$\mathrm{LOQ}=\frac{10S_{a}}{b}$$

Here, *S_a_* corresponds to the standard deviation of y-intercepts, while *b* represents the slope of the calibration curve. This approach, although demanding more data and effort, yields more accurate and practical LOD and LOQ values, ensuring the reliability of gas sensor measurements in real-world applications.

### 3.2. Reliability of the Experimental Conditions in Ultrafast Sensors

The second problem associated with ultrafast sensors raises crucial concerns about the reliability of experimental conditions. When researchers claim to detect a gas pollutant in mere seconds or milliseconds, several intricate issues come to the fore, challenging the accuracy and validity of these rapid responses.

**Figure 4:**
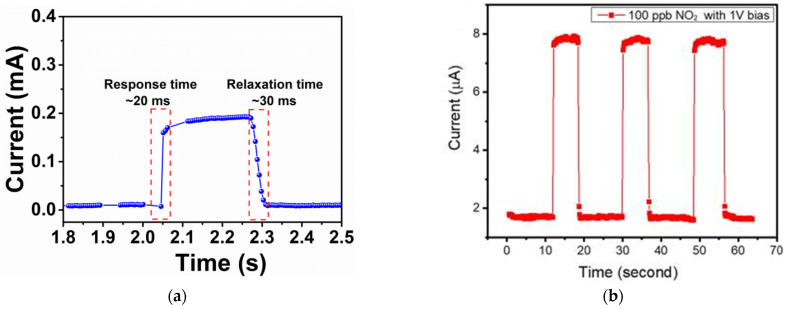
(**a**) Pressure sensor with response and relaxation times of 20 and 30 ms for 7 kPa, reproduced from [44], with permission from John Wiley & Sons; (**b**) gas sensor reporting response and recovery times of 50 and 75 ms when detecting 100 ppb of NO_2_, adapted from [45], with permission from the Royal Society of Chemistry.

(a) **Sensitivity to pressure changes**: A fundamental challenge arises from the extreme sensitivity of resistive sensors to pressure changes within the testing chamber [43], often induced by gas injection. Figure 4a illustrates this sensitivity by employing a piezoresistive nanomaterial [44], where even slight pressure alterations can result in sensor responses of a few milliseconds (ms). In this regard, Figure 4b reports an ultrafast gas sensor [45], claiming that injecting 100 ppb of NO_2_ led to a response and recovery times of 50 and 75 milliseconds, respectively. Considering that exposure limits are set in hours, detecting NO_2_ in milliseconds raises questions about the practicality and relevance of such rapid responses. Moreover, the rapid adsorption and desorption displayed challenges to standard physico–chemical rules, particularly if sensors operate under standard conditions. The absence of critical experimental parameters, such as flow rates, in publications like Figure 4b raises concerns. Achieving a homogeneous concentration of the target pollutant within milliseconds (50 ms in Figure 4b) is questionable and necessitates a deeper examination of experimental conditions.

(b) **Rapid and reversible physico–chemical reactions**: Ultrafast sensors claim remarkable speed and reversibility in physico–chemical reactions, presenting perplexing issues. Resistors typically exhibit relatively slow responses, especially at room or moderate temperatures. The astonishing speed of ultrafast sensors raises questions about the underlying mechanisms of these reactions. The rapid and reversible nature of these reactions demands meticulous scrutiny of the experimental setup to ensure the accuracy and reliability of the results. Indeed, Figure 4b illustrates another perplexing phenomenon: signal saturation leading to steady-state conditions in less than a second [45]. This rapid attainment of saturation raises intriguing questions about the underlying mechanisms governing ultrafast sensor responses. Achieving a stable state within such a short timeframe challenges conventional understanding and necessitates a thorough investigation into the physico–chemical processes at play.

(c) **Homogeneity of gas concentration**: Ensuring a uniform gas concentration within milliseconds or seconds in the testing chamber presents a significant challenge. Ultrafast sensors boast almost instantaneous gas detection, but achieving a uniformly distributed gas concentration in such a short time frame is technologically complex. Minor pressure variations due to an excessive flow rate or incorrect flow orientation [46,47,48] can lead to false positives, providing a misleading impression of rapid and sensitive gas detection.

Addressing these challenges requires a rigorous approach to experimental design and validation. Researchers must exercise meticulous control over and monitor pressure changes within the testing chamber. Understanding the intricacies of physico–chemical reactions and conducting experiments under diverse conditions is essential to ensuring the reliability and accuracy of ultrafast gas sensors in real-world applications. For instance, conducting longer exposures, spanning several minutes, is crucial. This duration allows researchers to dismiss the possibility of merely measuring pressure changes within the testing chamber, ensuring that the sensor responses genuinely correspond to gas interactions and not extraneous factors. Furthermore, understanding the intricate relationship between the flow rate and the chamber’s volume is paramount. This understanding is essential to unraveling the sensing response accurately and preventing false positives. Excessive flow rates can introduce turbulence, disrupting the gas distribution within the chamber. Similarly, an incorrect chamber design might lead to inconsistencies in the results. Therefore, careful consideration of these factors is indispensable in designing experiments that yield reliable and meaningful data from ultrafast sensors. By addressing these aspects, researchers can enhance the credibility and applicability of ultrafast gas sensor technology in various real-world scenarios.

## 4. Conclusions

Data from continuous monitoring serve as the basis for policy formulation and evaluation. Governments and regulatory bodies rely on these data to set air quality standards, develop emission controls, and assess the effectiveness of existing policies. Informed decision-making is impossible without a real-time understanding of the current state of the atmosphere. Ultimately, an extended sensing network, supported by a high density of measuring nodes, stands as a vital tool in our ongoing battle to preserve air quality, protect public health, and foster sustainable urban environments.

By monitoring these pollutants, scientists and policymakers can assess the environmental footprint of human activities, aiding in the development of strategies to mitigate climate change and protect human health. In essence, the constant monitoring of air pollutants is not merely a scientific endeavor; it is a societal responsibility that empowers people to make informed decisions. The continuous vigilance provided by air quality monitoring remains our most potent tool in creating a healthier and more sustainable future.

This study emphasizes the practical superiority of slower gas sensors in ambient monitoring contexts, a preference justified by several critical factors. The necessity for reliable time-averaged exposure assessments aligns with the slow sensors’ capability to provide accurate and representative measurements. Slow sensing offers additional advantages, including enhanced reliability, cost-effectiveness, and compatibility with regulations. These sensors provide more representative data by averaging out short-term fluctuations, reducing the likelihood of false positives and interferences, and offering improved signal-to-noise ratios. This contrasts with ultrafast sensors, which, while claiming swift responses, often compromise accuracy and reliability.

The importance of accurate quantification cannot be overstated. Ultrafast sensors might detect pollutants within seconds, but quantifying these detections accurately is an intricate challenge. Slow sensors, with their comparatively longer response times, offer a balanced approach, allowing for meticulous quantification and significantly better signal-to-noise ratios, thus ensuring the integrity of the data collected.

Furthermore, the experimental challenges faced by ultrafast sensors, such as sensitivity to pressure changes, rapid and reversible reactions, and the need for homogeneous gas distribution, cast serious doubts on their reliability. Slower sensors, on the other hand, provide stable measurements and are less prone to false positives and interferences. They ensure the integrity of the data by averaging out transient fluctuations and thus offering a more accurate representation of long-term exposure levels.

In practical terms, the benefits of slower gas sensors extend beyond just accuracy. Their cost-effectiveness, ease of implementation, and compatibility with regulations make them the ideal choice for ambient monitoring networks. Their simpler design and lower maintenance requirements ensure sustained, long-term monitoring efforts crucial for policy-making and public health initiatives. Thereby, slow gas sensors, often sidelined in the race for speed, emerge as the pragmatic choice for real-world applications. Their ability to provide accurate, reliable, and consistent data over time is invaluable, ensuring that our efforts towards mitigating air pollution are founded on robust and trustworthy information.

## Figures and Tables

**Table 1:** Most representative regulatory agencies and organizations for establishing the guidelines about gas exposure limits in different countries in 2023.

Country/Region	Abbreviation	Institution
European Union	EU-OSHA	European Agency for Safety and Health at Work
	ECHA	European Chemicals Agency
United States	OSHA	Occupational Safety and Health Administration
	NIOSH	National Institute for Occupational Safety and Health
China	SAMR	State Administration for Market Regulation
Japan	JSOH	Japan Society for Occupational Health
India	MOL&E	Ministry of Labour and Employment
United Kingdom	HSE	Health and Safety Executive
Canada	CCOHS	Canadian Centre for Occupational Health and Safety
Australia	SWA	Safe Work Australia
